# Supplementary material for: MicroRNAs Associated with Chronic Mucus Hypersecretion in COPD Are Involved in Fibroblast–Epithelium Crosstalk
Source: Cells. 2022 Feb 2;11(3):526. doi: 10.3390/cells11030526 (PMC8833971; doi:10.3390/cells11030526)
Supplement: Supplementary file 1 [file cells-11-00526-s001.zip › cells-1501964-supplementary.pdf]

MicroRNAs Associated with Chronic Mucus Hypersecretion in COPD Are Involved in Fibroblast–Epithelium Crosstalk

Hataitip Tasena [1,2,3,4], Wim Timens [1,2], Maarten van den Berge [2,5], Joy van Broekhuizen [1,2], Brian K. Kennedy [3,4,6,7], Machteld N. Hylkema [1,2,†], Corry-Anke Brandsma [1,2,†] and Irene H. Heijink [1,2,5,*]

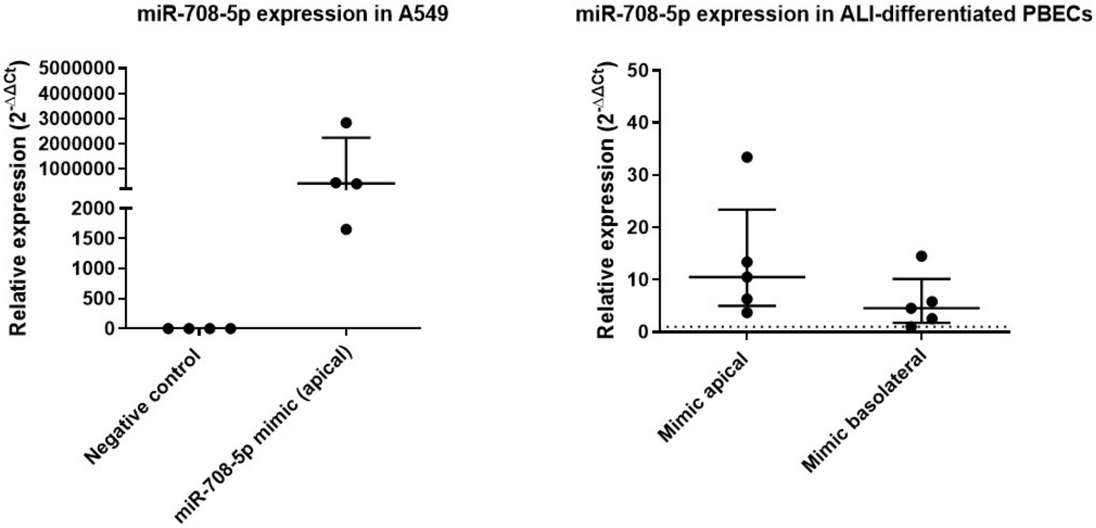

Figure S1. miR-708-5p expression upon transfection with miR-708-5p mimic in A549 and air-liquid interface (ALI)-cultured primary bronchial epithelial cells (PBECs). A549 cells or PBECs cultured at ALI for 14 days were transfected with miR-708-5p mimic either from the apical (1 nM) or the basolateral (3 nM) side. The expression of miR-708 was normalized to RNU48 and related to the negative control ($2^{-\Delta\Delta Ct}$). Four-five experiments were performed independently.

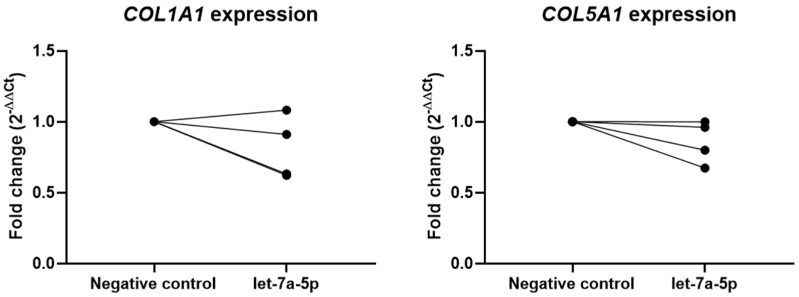

Figure S2. Effect of let-7a-5p overexpression on *COL1A1* and *COL5A1* expression in normal human lung fibroblasts (NHLFs). NHLFs were seeded in 24-well plates and transfected with let-7a-5p mimic or negative control siRNA (5 nM) were transfected in upon. RNA samples were collected 24 hours after the medium refreshment. The expression if *COL1A1* and *COL5A1* was normalized to *GAPDH*. Four experiments were performed independently.
